# Extracellular Vesicles and Their Current Role in Cancer Immunotherapy

**DOI:** 10.3390/cancers13092280

**Published:** 2021-05-10

**Authors:** Carla Giacobino, Marta Canta, Cristina Fornaguera, Salvador Borrós, Valentina Cauda

**Affiliations:** 1 Department of Applied Science and Technology, Politecnico di Torino, Corso Duca degli Abruzzi 24, 10129 Turin, Italy; s255203@studenti.polito.it (C.G.); marta.canta@polito.it (M.C.); 2 Grup d’Enginyeria de Materials (Gemat), Institut Químic de Sarrià (IQS), Universitat Ramon Llull (URL), Via Augusta 390, 08017 Barcelona, Spain; cristina.fornaguera@iqs.url.edu (C.F.); salvador.borros@iqs.url.edu (S.B.)

**Keywords:** extracellular vesicles, cancer vaccine, immunotherapy, drug delivery, gene delivery, surface funzionalization, nanoparticles

## Abstract

**Simple Summary:**

In recent years, immunotherapy has shown great advancement, becoming a powerful tool to combat cancer. In this context, the use of biologically derived vesicles has also acquired importance for cancer immunotherapy. Extracellular vesicles are thus proposed to transport molecules able to trigger an immune response and thus fight cancer cells. As a particular immunotherapeutic approach, a new technique also consists in the exploitation of extracellular vesicles as new cancer vaccines. The present review provides basic notions on cancer immunotherapy and describes several clinical trials in which therapeutic anticancer vaccines are tested. In particular, the potential of extracellular vesicles-based therapeutic vaccines in the treatment of cancer patients is highlighted, even with advanced stage-cancer. A focus on the clinical studies, already completed or still in progress, is offered and a systematic collection and reorganization of the present literature on this topic is proposed to the reader.

**Abstract:**

Extracellular vesicles (EVs) are natural particles formed by the lipid bilayer and released from almost all cell types to the extracellular environment both under physiological conditions and in presence of a disease. EVs are involved in many biological processes including intercellular communication, acting as natural carriers in the transfer of various biomolecules such as DNA, various RNA types, proteins and different phospholipids. Thanks to their transfer and targeting abilities, they can be employed in drug and gene delivery and have been proposed for the treatment of different diseases, including cancer. Recently, the use of EVs as biological carriers has also been extended to cancer immunotherapy. This new technique of cancer treatment involves the use of EVs to transport molecules capable of triggering an immune response to damage cancer cells. Several studies have analyzed the possibility of using EVs in new cancer vaccines, which represent a particular form of immunotherapy. In the literature there are only few publications that systematically group and collectively discuss these studies. Therefore, the purpose of this review is to illustrate and give a partial reorganization to what has been produced in the literature so far. We provide basic notions on cancer immunotherapy and describe some clinical trials in which therapeutic cancer vaccines are tested. We thus focus attention on the potential of EV-based therapeutic vaccines in the treatment of cancer patients, overviewing the clinically relevant trials, completed or still in progress, which open up new perspectives in the fight against cancer.

## 1. Extracellular Vesicles: An Introduction

**Figure 1:**
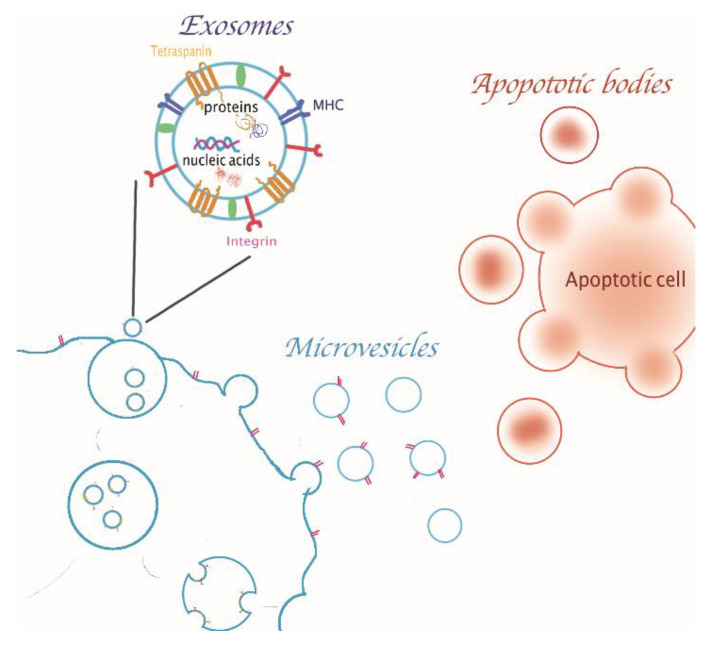
Extracellular vesicles biogenesis and a specific focus on exosome composition. Exosomes originate intracellularly as intraluminal vesicles of the multivesicular bodies; microvesicles originate from the outwards budding and fission of plasma membrane; apoptotic bodies are caused by the fragmentation of the apoptotic cells. The exosomes membrane is composed by different kinds of lipids and proteins and they can carry inside cytosol-derived molecules, such as proteins and nucleic acids.

According to the latest literature reports, extracellular vesicles (EVs) are particles with a spherical shape and delimited by a phospholipid bilayer. They are produced by several cell types according to different physiological processes and even pathological conditions and released into the extracellular environment [1]. EVs can be thus extracted from fluids of biological origin, such as blood, saliva, urine, amniotic fluid, breast milk, cerebrospinal fluid, and synovial fluid [2]. Although EVs were initially considered to be part of the waste management of the cells [3], many advancements have been achieved so far to unravel their mechanisms of the transfer of cell-derived biomolecules (i.e., DNA, various RNA types, proteins, lipids and metabolites), characterizing the communication between different cells and tissues [4]. For this reason, in recent years, more and more attention has been paid to extracellular vesicles and many research and literature reviews have emerged so far [1,2,3,4,5,6]. Furthermore, EVs are also involved in processes such as angiogenesis, coagulation, cell survival, waste management, immunomodulation and inflammation [5]. The International Society for Extracellular Vesicles (ISEV) suggests the term “extracellular vesicle” to define particles delimited by a lipid bilayer, naturally released by cells and unable to replicate. The lipid bilayer membrane of EVs protects their load from enzymatic degradation during the transfer from donor to recipient cells [3]. Packaging also allows cargo to be stored more efficiently and to deliver it at dedicated target cells by modifying the vesicles with cell type-specific adhesion receptors. For this reason, new clinical applications of EVs for therapy or as biomarkers reporting about healthy and diseased conditions are under development, rising increasing interest in both the research and clinical community [6]. However, due to their heterogeneity, at the moment the definition of EVs subclasses cannot be based on their specific markers expressed at EVs surface. Anyhow, it is possible to classify them into three groups, according to their size and biogenesis’ mechanism: microvesicles, apoptotic bodies and exosomes [7]. Figure 1 illustrates the different EVs biogenesis mechanisms and an example of exosome composition.

Microvesicles (MVs) are heterogeneous cell-derived membrane vesicles that protrude from the surface of cells in a highly regulated process and are then released to the extracellular environment [8]. They are large vesicles with a diameter from 100–1000 nm, detected in many bodily fluids, such as blood, urine, synovial fluid, and many others, under both physiological and pathological conditions. Moreover, elevated MV concentrations have been also observed in atherosclerotic plaques and tumor tissues [9]. In general, MVs are the result of the budding of the cell membrane from which they take shape through ARF6-mediated (ADP-ribosylation factor 6) rearrangement of the actin cytoskeleton. The mechanisms of formation and release of MVs remain only partially understood. Microvesicles biogenesis also involves the trafficking of cargo molecules towards the cell membrane and the redistribution of phospholipids of the plasma membrane. The specific function of microvesicles is determined by the composition of their molecular cargo, which, from what described above, is in turn clearly dependent upon the progenitor cell type and condition, as well as upon the microenvironment and the triggering stimuli which preceded the MVs release. Generally, microvesicles transport membrane-derived receptors, cytokines, chemokines, proteins involved in cellular signaling, lipids, carbohydrates [2], and nucleic acids, such as DNA and various types of RNA, including mRNAs, microRNAs (miRNAs), and small-interfering RNAs (siRNAs) [9]. When microvesicles are released into the extracellular environment, their cargo can be released and used to modify or alter the extracellular milieu. In other cases, MVs can dock to recipient cells and be internalized via endocytosis or fusion mechanisms, or even activate a signaling cascade mediated by the cell receptors [8]. Therefore, MVs are also fundamental in modifying the extracellular environment and signaling among cells, in targeting specific recipient cells, as well as in assisting the cell invasion and metastasis towards tissues using a cell-independent matrix proteolysis mechanism, or even in transferring specific receptors to recipient cells, thus enabling new cell signaling in cells initially missing the receptor. In the end, the MVs, while taking part in cellular communication, affect processes such as blood coagulation, thrombosis, angiogenesis, immunomodulation and inflammation [2]. 

Apoptotic bodies (ApoBDs) are vesicles bound to cell membrane, ranging from 50–5000 nm in diameter, which are then released from cells undergoing apoptosis [4]. Apoptosis is a physiologically-programmed cell death that does not induce inflammatory responses. It is commonly appearing in multicellular organisms as it consists in a homeostatic mechanism for controlling the population of cells in a tissue and has a key role during the processes of development and aging. The splitting of cellular content through the membrane blebbing determines the formation of distinct membrane-delimited vesicles: the apoptotic bodies. These apoptotic bodies are then engulfed by phagocytes for final degradation [10]. This process is immunologically silent, which is a typical characteristic of apoptosis. Different cell types can use different mechanisms to disassemble and thus to undergo in apoptosis. Thus, apoptotic bodies can vary their content and biomolecules such as glycosylated proteins, chromatin, large amounts of RNA, nuclear fragments, and even intact organelles (mitochondria) can be included. Despite this diversity in the apoptotic mechanism depending to the cell type, recent reports have shown that ApoBDs are also involved in the formation and conditioning of the tumor microenvironment and metastatic niche. It has been understood that this mechanism lies in the active role of ApoBDs to transfer biomolecules towards specific “target” cells [11]. Furthermore, ApoBDs have been also reported to show a consistent procoagulant effect towards cancer cells, which in turn would contribute to the prothrombotic state and immune regulation. Actually, the formation process of apoptotic body is closely related to cell death and clearance, and ultimately in the intercellular communication [12]. These mechanisms have direct implications in the regulation of immune system, which is particularly relevant in cancer therapy. Therefore, ApoBDs can promote an important role in anticancer immunity and further studies are needed to clearly demonstrate their role and further use in medicine. 

Exosomes are vesicles with a diameter of 40–100 nm and derive from cell secretion upon multivesicular bodies (MVBs) formation [13]. Exosomes are released by almost all cell types and, as the other above-mentioned EVs, are present in many different bodily fluids. Exosome also have a spherical shape and a nanosized dimension, and their phospholipid bilayer membrane is composed of different phospholipids and proteins types (including transport proteins, heat shock proteins, tetraspanins) [14], which in turn derive from the cell of origin. In particular, according to recent databases (ExoCarta and EVPedia), it results that the protein composition of exosomes is somehow defined and include both conserved and cell-specific proteins. Interestingly, the tetraspanins CD9, CD63, CD37, CD81, or CD82 are typically present in the exosomes membrane and thus used as biomarkers for exosome identification [15]. 

Exosomes were identified in extracellular space for the first time in late 1980s. They were initially considered as cellular waste or as by-products of cell homeostasis [16]. Currently, these extracellular vesicles are considered functional vehicles, because they are able to deliver molecular cargoes to target cells and reprogram the behavior of recipient cells even located far from exosome release site. Generally, exosomes contain proteins, DNA, mRNA, miRNA, lipids, and this molecular composition directly derives from the parent cell, reflecting clearly the signature of the multiple physiological roles or pathological state of the progenitor cell. It is broadly recognized that exosomes play a major role in intercellular communication, but they also take part in many biological processes, including antigen presentation in immune responses, coagulation, inflammation, maturation of erythrocytes, and angiogenesis [14]. It has been recently discovered the role of exosomes in tumor progression and metastasis formation, in particular in pre-conditioning of the metastatic niche and tumor microenvironment. In this sense, exosomes are responsible of transferring bioactive molecules from the primary tumor site to other cells and tissue, both in the local and distant microenvironments [17]. 

In the recent years, the use of vesicles as biological carriers has also been extended to cancer immunotherapy [18]. This kind of cancer treatment involves the use of extracellular vesicles to transport molecules capable of triggering an immune response to damage cancer cells. In particular, different literature reports have studied the possibility of using extracellular vesicles as new cancer vaccines, which represent a particular form of immunotherapy. However, in the literature there are only few publications that systematically gather and collectively discuss these studies. A recent review [19] highlights specifically the role of exosome, a subclass of EVs, as mediators of immune regulation of both lymphoid and myeloid cells in cancer. However, in that review and others present in the literature, the re-engineering of EVs to become therapeutic players for immunotherapy against cancer is poorly highlighted. 

Therefore, the purpose and the novelty of our review is to illustrate and give a reorganization to what has been produced in the literature so far [20], related to both the research and the clinical studies made on therapeutic anticancer vaccines based on extracellular vesicles, completed or still in progress.

The review begins by a defining extracellular vesicles and highlighting importance concepts related to their therapeutic potential. In view of many and prominent reviews present in the literature to date [1,2,3,4,5,6,14,16,17], here we skip on the detailed the discussion about the EVs nature, origin and types. For the same reason, the description of cargo loading techniques for EVs are only briefly introduced, being recently reported already [21]. Some engineering techniques, aimed at introducing surface markers for cell targeting, are however reported here with the aim to give a proper outline of the main concepts related to extracellular vesicles modifications and re-engineering. We specifically provide the reader with basic notions about cancer immunotherapy and show several examples, including clinical trials, in which therapeutic anticancer vaccines are involved. We then focus on the potential role of EVs as therapeutic vaccines in the treatment of cancer patients, even with advanced stage-cancers, focusing on clinical trials. Thus, the novel perspective of the present review is not only the discussion of the interaction between EVs and immunology, but to report on the biological applications of re-engineered EVs as cancer vaccines.

## 2. EVs for Therapeutic and Drug Delivery Purposes

EVs have various advantages such low immunogenicity, toxicity and targeting ability. Furthermore, the use of EVs can overcome some limitations encountered in conventional nanoparticulate systems used for drug delivery, such as liposomes, which are vesicular structures prepared from lipids in the laboratory and widely used as drug carriers. Unlike liposomes, exosomes-based delivery systems pass through main biological barriers, such as the blood brain barrier (BBB) [22,23], evade the lysosomal degradation and transport cargoes into the cytoplasm [24]. Therefore, they are ideal candidates for constructing novel therapeutic delivery nanosystems.

Thanks to new technologies enabling for isolating microvesicles and their ability to deliver and transfer biomolecules such as nucleic acids, also microvesicles can be exploited for targeted and therapeutic drug delivery, which can be further improved by engineering the microvesicles. The first study on the delivery of mRNA and proteins through MVs for cancer therapeutics has indeed shown that such genetically engineered vesicles are viable delivery vehicles. In particular the authors showed the ability to deliver suicide mRNA genes to cancerous schwannoma cells [25]. MVs were isolated from cells which stably expressed the suicide gene of interest and a protein–cytosine deaminase (CD) fused to uracil phosphoribosyltransferase (UPRT), which constitutes a valid prodrug-activating combination. Such MVs were then directly injected into schwannoma tumor of an orthotopic mouse model in combination the prodrug (5-fluorocytosine, 5-FC), which is then transformed within the tumor cell to 5-fluorouracil (5-FU), an anticancer agent. Therefore, the combination treatment of CD-UPRT mRNA/protein via MVs and 5-FU led to regression of these tumors. Recently, a group of researchers from Michigan State University (USA) demonstrated the efficacy of using MVs as a delivery vehicle in cancer treatment in a breast cancer mouse model [26]. They loaded MVs with engineered minicircle plasmid DNA encoding a thymidine kinase fusion protein. Such protein is then able to activate two prodrugs, ganciclovir and CB1954, against breast cancer cells. Then, they detected that the efficiency of delivery of MVs loaded with the engineered DNA was 14-times greater than the efficacy given by MVs loaded with regular plasmids. In addition, minicircle-loaded MVs were even more successful in destroying cancer cells. These outcomes confirm that gene delivery via MVs enables an effective drug delivery, so it can be considered an alternative to chemotherapy and, thanks to the high compatibility with the human body, unwanted immune responses can also be reduced.

The natural role of exosomes in cell communication, as well as their unique characteristics, enable them to be ideal candidates as drug delivery systems, promoting their application in both drugs and biomolecules delivery. Indeed, other than having a low immunogenicity and toxicity, these vesicles are able to transport a great variety of substances both in their core or associated to their membrane.

Exosomes are secreted by many different cell types, each influencing their nature and biological role. From the proteomic analysis it emerges that other than the ubiquitous proteins (f.i. tetraspanin, alix, TSG101), exosomes are composed by cell type-specific proteins inherited by their cellular source that condition their biological activities. For instance, exosomes released by mature reticulocytes show elevated levels of transferrin receptors, that is lost by these cells during their maturation. Similarly, the aquaporin proteins, involved in the water transport, are enriched in exosomes derived from kidney cells [24,27].

Other two important factors that promote the application of exosomes as new delivery systems are the amenability to modifications, thus to enhance exosomal targeting capability and the scalability of the process. Generally, it is possible to introduce modifications to the exosomal membrane proteins which are responsible for cell targeting. However, exosomes derived from specific cell source have been investigated to solve the problem of cell targeting or to produce a desirable therapeutic effect. Some exosomes released by tumor cells are reported harbouring an intrinsic targeting activity versus the tumor site that could be exploited in clinical applications. Other surface specific proteins, called tumor associated antigens, could be transferred to dendritic cells (DC), thus used to promote an immune reaction against cancer cells [28].

Therefore, the delivery ability of these kind of vesicles has already opened new scenarios for the development of new exosomes-based therapeutic agents and also for their application in cancer immunotherapy.

Cancer immunotherapy is defined as “the artificial stimulation of the immune system to treat cancer” [29]. This cancer treatment approach is based on the idea to use the immune system to fight and destroy the cancer cells, exploiting the natural immune mechanisms already normally used by the immune system to defeat diseases. Indeed, EVs could play a key role in immune regulation of cancer development through the use of those EVs released by immune cells or cancer cells. There is actually a controversy on the most suitable exosomes donor cells to be used, due to their putative involvement in immune system activation against cancer but also as immunesupressors, as reoprted already for glioblastoma, pancreatic and ovarian cancers [30,31,32]. In addition, cancer derived exosomes arise also some controversy due to their origin from malignant cells. However, upon engineering, there is no doubt on their beneficial use given that the antibodies produced by the immune system could be trained to bind to the tumor’s antigens, identifying in this way the cancer cells and promoting their elimination. However, at the same time, tumor-derived exosomes could produce undesired effects, such as the inhibition or the killing of the cytotoxic T cells, used by the immune system to destroy its enemies. As a result, the release of exosomes by tumors may allow them to evade the “immunosurveillance” and interfere with cancer immunotherapy [33,34]. The potential risk in using tumor exosomes and the possibility in aggravating the pathological condition of the patients, induced the scientific community to explore new exosomes sources. Exosomes derived from plants or agricultural products, such as milk, have been proved to be a most safe and really scalable options to produce exosomes for clinical applications [35]. However, in the context of cancer immunotherapy they are not the best choice. In fact, unlike those derived from tumors, these are unable to stimulate the immune system for the cancer treatment.

As a consequence, immune cell-derived exosomes have been investigated, in particular those derived from DC cells [36,37]. These cells are main components of the immune systems and act as antigen presenting cells to the T effector cells [38]. Consequently, DC-derived exosomes contain antigen presenting molecules, adhesion molecules and costimulatory molecules, that are the necessary equipment required for generating powerful immune responses and thus for exosome-based vaccines. This new technique for the therapeutic administration of the vaccines is based on the production of exosomes, engineered with the vaccine antigen of interest, to induce a powerful cytotoxic T cell (CTL) mediated immune response against a large number of tumors and viral antigens. Other than the DC cells, another attractive healthy exosomes’ source are the Mesenchymal Stem Cells (MSC). These cells are able to self-renewal and are multipotent since they can differentiate in different kind of cells and also own many useful characteristics [39]. They could be isolated from many different sources (f.i. bone marrow, placenta, umbilical cord), they are easily expanded In vitro to produce large amounts of exosomes and they harbour immunosuppressive properties that are demonstrated transferable to their exosomes [40]. The only main limitation associated to these cells is that they are not immortal, so the number of produced exosomes is limited by the number of their replications. Yeo et al. [27] investigated the immortalization of human embryonic stem cells (hESC-MSC) with the oncogene *MYC* to avoid this limitation, allowing the growth of this kind of cells In vitro for even long periods. Similar immortalization approaches have been reporte so far, proposing a feasible manufacturing method for therapeutic EVs [41,42].

Another, yet to be developed, application of exosomes, could be in enhancing the performance of CAR-T cell therapies, given the natural role of exosomes to activate T cells through antigen presentation. Although some studies previously reported the possible potent antitumor effect of exosomes isolated from CAR-T cells [43], bibliography is still scarce and many efforts will be required to achieve this specific application transference to clinical use.

## 3. Cargo-Loading Methods of EVs

The unique structure of EV membrane, constituted by a phospholipid bilayer with a hydrophobic space within the bilayer and a hydrophilic surface, allows a great variety of molecules to be loaded into the EVs [3,21]. Hydrophobic and hydrophilic molecules can be loaded into EVs, including anticancer drugs, miRNA, siRNA, DNA and proteins additional to their physiological molecular composition [35,44,45]. Recently, EVs have been proposed to carry nanoparticles (NPs) as well [46,47,48,49]. By loading NPs into EVs, it is possible to overcome problems such as particle aggregation, degradation and rapid clearance, which often occur in the use of nanoparticles.

Therapeutic cargos are incorporated into EVs by following two main loading approaches: exogenous (or direct loading) and endogenous loading approaches [3,21]. By exogenous methods, therapeutic cargos are loaded into EVs after their isolation. In addition, these techniques are subclassified into passive and active cargo-loading methods. Passive loading refers to a simple method wherein cargo is passively loaded into EVs without any external interventions. Instead, active loading uses different techniques that force EVs to load the cargo. Cargo-loading methods of EVs have been illustrated in other more detailed reviews [1,3,21] and will thus be no further discussed here.

Endogenous loading refers to (a) genetic engineering of donor cells to constitutively produce exosomes loaded with the Active Pharmaceutical Ingredients (API) of interest, or to (b) transient donor cells transfection to achieve the release of loaded exosomes. In particular, some genetic engineering techniques are used for surface functionalization and are briefly described below in Section 4.

## 4. EVs Surface Functionalization: An Overview

The functionalization of the EVs surface is carried out to improve targeting abilities, biodistribution and therapeutic applications of EVs. However, the use of this approach needs the development of protocols with a strict control of the experimental conditions (f.i. temperature, pressure, solvents, salt concentrations) to preserve the exosomes integrity and functions. Indeed, undesired effects for therapeutic applications, such as vesicles aggregation due to inadequate reaction conditions, have been reported in the literature so far [50].

### 4.1. Post-Isolation Methods

Between the methods used for the EVs surface modifications there are the covalent and non-covalent chemical modifications [1]. The non-covalent approaches provide membrane modifications through the use of gentle reactions, such as the electrostatic interactions or the hydrophobic insertions [50]. For this purpose, Nakase et al. [51] used Lipofectamine, a commercial transfection reagent containing cationic lipids, to modify the EVs charge and promote their interaction with the plasma membrane. Indeed, the adsorption of the Lipofectamine on the EVs surface confers them a positive charge helping their interaction with the negative domains of the target cell membrane. In addition, the functionalization with cationic lipids was used to recruit the negatively charged fusogenic peptide GALA. This peptide promoted the EVs escape from the endosomal compartments, essential for many drug delivery applications [52]. Therefore, as it is evident, the functionalization based on electrostatic interaction combined with the application of the GALA peptide can be used to improve cellular uptake and also enhance the exosomal escape and content release in the cytosolic space.

The covalent methods, on the contrary, need the formation of new chemical bonds at the EVs surface. A widely diffused method is the click chemistry, that adds molecules on the EVs surface through a cicloaddiction reaction. This method can be easily performed and is highly versatile in terms of reaction conditions, such as pH and temperature ranges [53]. Furthermore, a study demonstrated that click chemistry did not alter exosome size and functionality [54]. Another common approach of surface functionalization of EVs, based on covalent bonding, is the PEGylation, to achieve stealth surfaces, as performed everywhere for different kinds of nanosystems. In fact, the addition of the polyethylene glycol (PEG) on the EV surfaces forms a corona that is effective in reducing their immunogenicity. A study by Kooijmans et al. [55] showed that this surface modification significantly increases the EV circulation half-life in mice because it reduces EVs recognition by the mononuclear phagocyte system (MPS) avoiding plasma protein opsonization. However, the presence of this PEG corona around the EVs surface is also been associated to a reduction in EVs interaction with the plasma membrane, thus a reduction of cellular uptake. To overcome these limitations, many authors functionalize the PEG with targeting ligands, directed to receptors overexpressed by the target cells [55,56].

For example, Kim et al. [57] loaded exosome with paclitaxel (PTX), an anti-cancer agent widely used against lung cancer, to be delivered to pulmonary metastases. They developed a specific procedure based on sonication and incubation to add PEG, and a similar procedure to incorporate a vector moiety with Aminoethylanisamide AA, a ligand of the sigma receptor, overexpressed by lung cells [58]. Experimental results showed that the AA-PEG-exoPTX formulation can be easily internalized into target cancer cells, other than a high loading capacity and strong anticancer effects compared to the unfunctionalized exosomes.

### 4.2. Genetic Engineering of Parental Cells for Surface Functionalization

The EVs surface can be functionalized with ligands not only through EV post-isolation methods such as click chemistry, but also through genetic engineering, wherein the cells that will produce the EVs are induced to express the protein or peptide of interest. This approach was employed by Alvarez-Erviti et al. [22] that used exosomes loaded with small interfering RNAs (siRNAs) to achieve knockdown of β-site APP-cleaving enzyme 1 (BACE 1), a therapeutic target for Alzheimer’s disease [59]. In particular, to obtain a selective targeting of this EVs versus the mouse brain, they functionalized the EVs with the targeting peptide obtained from Rabies Virus Glycoprotein (RVG), able to bind the nicotinic acetyl choline receptor (AchR) highly expressed by the cells at the blood brain barrier level [13]. This functionalization was obtained attaching this peptide to the Lamp2b protein expressed on the EVs surface, transfecting the cell with the plasmid codifying for this construct. Moreover, researchers observed a strong knockdown of BACE 1 and an important reduction of amyloid plaques components, which are in turn associated to Alzheimer’s pathology. In a recent study, Yang et al. [60] have found a similar approach to systematically deliver nerve growth factor (NGF) into ischemic cortex for the treatment of stroke, in a photothrombotic ischemia model. NGF has a primary role in the growth, as well as the maintenance, proliferation, and survival of nerve cells [61]. The exosomes loaded with the mRNA coding for the NGF protein were functionalized on their surface with the peptide RVG, thus obtaining NGF@ExoRVG. The in vivo administration of such modified exosomes led to an effective delivery of the NGF mRNA to the cells of the ischemic cortex and in its consequent protein translation. Interestingly, the treatment with these exosomes was associated to a reduced inflammation mediated by the M2 microglia: a process which mediates the immune response in the CNS.

All these results suggest that the exosomes engineering could be a useful tool for the production of exosome-based delivery vehicles, providing new options for target therapies.

## 5. Cancer Immunotherapy

Immune system is a defensive apparatus that the human body uses to fight illness. It is constituted by a complex “surveillance network” made up of several highly specialized organs and cells, shared by the lymphatic vessels, and located in various parts of the body. All of them cooperate, each with a specific role, to defend the organism and keep it healthy. The immune system has the main role to trace the “foreign” substances present in the organism. Therefore, whenever substance not normally present in the organism enters the body triggers an alarm and causes an immune response, aimed at destroying it. Unfortunately, cancer can commonly escape the immune system’s natural defences, allowing cancer cells to continue growing. Immunotherapy is specialized in the development of novel anti-cancer therapies by understanding and making use of immune pathways. This innovative cancer treatment stimulates the natural immune system to fight cancer by finding and attacking cancer cells. There are many types of immunotherapy including monoclonal antibodies, oncolytic virus therapy, adoptive cell therapies and cancer vaccines.

Monoclonal antibodies are highly specific molecules produced in laboratory from identical immune cells and engineered to work as substitute antibodies that target only a single site (epitope) on a single antigen in order to obtain a strong immune response against cancer cells [62]. The monoclonal antibody recognizes the presence of a specific receptor on the surface of the tumor cell and binds to it. In this way, it induces the immune system to attack and destroy cancer cells while minimizing the damage to healthy cells. It can also induce cancer cells to self-destruct or can block the receptor preventing it from binding to a different protein that stimulates the cancer growth. Some examples of commercialized monoclonal antibodies for cancer treatment or maintenance cancer therapy are trastuzumab and pertuzumab for breast cancer and rituximab for non-Hodgkins lymphoma.

The goal of oncolytic virus therapy is to use viruses to destroy tumors; in particular, the oncolytic viruses (OVs) are able to replicate in cancer cells (while not in healthy ones) producing a lysis of the tumor mass. At the same time, OVs can stimulate the immune system to implement a strong and lasting response against the tumor itself. Unfortunately, it is necessary to consider that this kind of therapy could be hindered by the possible occurrence of an anti-viral response due to the recognition of the virus as a pathogen. Therefore, many researchers are currently looking for a solution that allows a right balance between anti-tumor and anti-viral immunity to make OV therapy as successful as possible [63].

Adoptive cell therapy requires that the patient’s autologous T cells be genetically engineered in the laboratory so that they express a receptor, called chimeric antigen receptor (CAR) specific for a tumor antigen. T cells are taken from a patient’s blood, then the gene for CAR expression is added to the T cells in the laboratory. After ex vivo cell expansion, CAR T cells are administered to the patient by infusion, then they bind the antigen on the cancer cells and kill them [64].

Lastly, cancer vaccines are designed to stimulate the immune system to recognize cancer cells in the patient and thus fight cancer more effectively [65].

The next section will investigate cancer vaccines more in detail and in particular the role of extracellular vesicles as delivery vehicles to enhance and amplify the effect of the immune response against tumor cells.

## 6. What Is a Cancer Vaccine?

Vaccines are generally prophylactic agents which are administered to healthy people to achieve long-term immunity against a virus and to prevent the consequent onset of the disease. However, cancer vaccines are different from prophylactic antiviral vaccines as they include not only (i) prophylactic vaccines used for cancer prevention, but also (ii) therapeutic vaccines [66]. Actually, currently existing prophylactic cancer vaccines only apply to virus-induced malignancies, such as liver cancer caused by the hepatitis B virus or genital cancers caused by the human papilloma virus (HPV). In contrast, therapeutic vaccines are designed for individuals with an existing disease. There are two aspects to take into account. First, cancer is often a silent disease and for this reason, at the time of diagnosis, it is already out of the control of the immune system and therefore already established in the body. Secondly, the vast majority of tumors that are classically treated with surgery, chemotherapy or radiotherapy definitively disappears. In some patients, however, the tumor may relapse and become increasingly resistant to treatment [67,68,69]. This happens because in some cases a certain number of cancer cells can escape treatment and remain in the body, even if the anticancer therapy was initially very effective. These cells are able to reproduce the tumor even years after its first appearance, and consequently the disease becomes more difficult to eradicate with classic treatments. In this context, recent clinical trials propose the combined or sequential use of different therapeutic strategies to create a “multimodal” therapeutic approach [70]. For example, therapeutic cancer vaccines are administered after complete surgical removal of the tumor, followed or not by chemotherapy, in lung cancer or cutaneous melanoma patients (NCT00530634, NCT04245514, NCT02211131, NCT04330430). The main goal of therapeutic vaccines is to elicit strong antigen-specific T cell responses, particularly CD8+ cytotoxic T lymphocytes (CTLs) mediated responses, with the assistance of suitable adjuvants which enhance the immune response [71]. CTLs are primarily responsible for the recognition and suppression of tumor cells: they recognize tumor-associated antigenic epitopes that are expressed by human leukocyte antigen (HLA) class I molecules on tumor cells, then attack, proliferate and cause cancer cell lysis [72]. The making of a cancer vaccine is based on the fundamental choice of the antigen together with its characteristics. The ideal antigen must be expressed only by tumor cells and not by normal cells; it must be present on all tumor cells in such a way that the cancer does not escape immune attack due to antigen downregulation; it must be highly immunogenic [73]. Antigens meeting all of these criteria do not exist, but they can be classified as (i) tumor-associated antigens (TAAs) and (ii) tumor-specific antigens (TSAs).

TAAs are self-antigens that can be expressed by both tumor cells and normal cells. However, the use of these antigens presents a major obstacle as the T lymphocytes that bind to TAAs are typically cleared by immune tolerance mechanisms. Immune tolerance is an important means by which growing tumors manipulate the tumor microenvironment with the aim of preventing elimination by the host immune system. Tolerance is the result of the so-called immune checkpoints. These pathways include the presence of protein receptors on the surface of immune system cells, which, if bound to specific ligands, prevent the immune system from attacking cells indiscriminately. Often, these specific ligands are expressed by tumor cells. In this way, cancer cells are able to trigger inhibitory signals that make immune cells inert or tolerant. For example, the binding between the checkpoint proteins PD-1 on the surface of T cells and the PD-L1 inhibitory receptors on tumor cells not only prevents T cells from attacking malignant cells in the body, but also inhibits T cells proliferation [74,75]. Therefore, to be effective, a cancer vaccine based on TAAs must be able to contrast and interrupt the tolerance mechanisms described above. Moreover, many vaccine clinical studies have revealed that the triggered immune response does not have significant efficacy. Indeed, Hollingsworth and Jansen [73] explain that these vaccines allow a very low rate of activation and proliferation of CD 8 T cells, compared to the amounts typically obtained with antiviral vaccines.

Conversely, TSAs, or tumor neoantigens, are truly tumor-specific as they are expressed only by cancer cells, highly immunogenic and dependent on tumor type [76]. Most importantly, these immunogenic neoantigens are the result of hotspot mutations which occur in numerous cancer patients so they are unique to each patient. For this reason, the design of a cancer vaccine based on single neoantigens requires a patient-specific approach. It starts with the sequencing of the tumor genome, then the mutations are identified and at the end, through computerized algorithms, the neoantigens are designed, possibly with experimental confirmation [73].

Antigens to be employed in therapeutic vaccines can be given to patients in the form of peptide-restricted epitopes, proteins, heat shock proteins transporting immunogenic peptides, recombinant viruses, autologous or allogeneic tumor cells, or DNA constructs. The main routes of administration are the direct injection, the coupling to immunostimulatory adjuvants and the ex-vivo loading of antigen presenting cells (APCs), typically dendritic cells (DCs) [72,77].

A peptide-based vaccine consists of immunogenic restricted epitopes, usually from tumor-specific or tumor-associated antigens, typically conjugated to a carrier protein [78]. The binding with the carrier protein helps enhance immune response thanks to its immunogenic properties and its characteristic of increasing the half-life of the epitope. Cancer cells can be distinguished from healthy cells because most of them are capable of overexpressing proteins or are characterized by mutations of those same proteins. Therefore, any gene product that has a mutated form with respect to healthy cells can be a potential target for the vaccine, just like the HER2 gene and its modified form HER2/neu that is one of the most studied oncogenes in cancer. HER2/neu is an epidermal growth factor (EGF) receptor. Human EGF is a protein naturally produced by the human body, which when attached to another protein, such as HER2 or CerbB2, stimulates the multiplication of cancer cells. About 30% of breast cancers develop along with the amplification of the HER2/neu gene or the overexpression of its protein product [79]. Its overexpression also takes place in other cancers such as stomach, ovarian, and colorectal cancers and in aggressive forms of pancreatic cancer, uterine cancer, and non-small cell lung cancer. Tumors characterized by HER2 overexpression are known as HER2-positive and can be highly aggressive [80]. Several peptide-based vaccines derived from the HER2 receptor have been developed in the last few years. NeuVax^TM^ is a 9-aminoacid peptide resulting from the combination of the extracellular domain of HER2 with granulocyte-macrophage colony-stimulating factor (GM-CSF). The vaccine stimulates specific CD8+ cytotoxic T lymphocytes to destroy HER2 positive cancer cells by promoting the lysis of breast cancer cells. Actually, Brossart et al. demonstrated that CTLs are also able to lyse other types of cancer cells expressing the HER2/neu oncogene, such as in the case of colon carcinoma and renal cell carcinoma [81]. Initial clinical trials showed that NeuVax^TM^ was well tolerated by patients but had no significant effect in preventing recurrence (NCT01479244). Currently, two Phase II clinical trials are examining NeuVax^TM^ treatment combined with trastuzumab in HER2-positive breast cancer to evaluate the real effectiveness of a combination therapy (NCT01570036, NCT02297698). Trastuzumab works by interfering with one of the ways in which breast cancer cells grow and divide. In particular, it attaches to the HER2 protein, thereby preventing the human growth factor in the epidermis from reaching the neoplastic cells and, consequently, preventing their division and growth. Trastuzumab also acts as a stimulator of the immune cells to improve their killing action against cancer cells. The main objectives of these two trials are disease-free survival at 24 months and 36 months and invasive disease-free survival from the beginning of therapy until the end of the study (5 years). Only NCT01570036 has been completed so far, by also achieving promising results in terms of rate of survival: disease-free survival as percentage of participants who survived at 24 months was 89.8%, at 36 months was 86.7%.

GP2 is another 9-amino acid peptide derived from HER2. Likewise, for this vaccine the preliminary In vitro tests have confirmed the ability to induce CTLs immune responses. Clinical studies have also shown that it is well tolerated and have also found an increase in HER2-specific CTLs. These are just two of the peptide-based vaccines designed specifically for breast cancer treatment [78]. It is also important to mention another peptide-based vaccine, HerVaxx. It consists of three peptides isolated from HER2 extracellular domain and linked to another carrier protein that is diphtheria toxin [82]. In the last few months a Phase 2 HerVaxx study was updated to evaluate the overall survival and the progression-free survival and to measure the efficacy, safety and immune response in 68 patients with metastatic gastric cancer overexpressing the HER-2 protein (NCT02795988).

In the examples described so far, the vaccine is directly injected to the patient. As already mentioned, however, it is possible to administer the vaccine also through the ex vivo loading of DCs. DCs are specialized antigen presenting cells (APCs) fundamental to the proper behavior of the immune system because they play as mediators between innate and adaptive immune responses. In particular, they act as sentinels throughout the organism and are able to recognize antigens. At the same time, through the processing of the antigenic material and the so-called “antigen presentation”, they allow the activation of the immune response implemented by the T lymphocytes. More precisely, DCs activate T lymphocytes through major histocompatibility complex (MHC) signaling [83]. These properties led to many attempts in the development of DC-based vaccines, achieving also promising results in individuals with advanced-stage cancers [84]. In 2010, the FDA approved the sipuleucel-T (PROVENGE^®^) for metastatic castration-resistant prostate cancer (mCRPC) [85]. Sipuleucel-T is an autologous DC vaccine based on enriched blood APCs cultured with a recombinant protein derived from the combination of prostatic acid phosphatase (PAP) with granulocyte-macrophage colony-stimulating factor (GM-CSF), that are, respectively, an antigen expressed in prostate cancer tissue and an immune cell activator [86]. Sipuleucel-T is a personalized therapy: DCs are directly taken from the peripheral blood mononuclear cells (PBMC) of the patient. Then, after ex vivo loading of PAP and activation, the vaccine is administered to the patient by infusion. In 2010, a Phase III clinical trial enrolled 512 mCRPC patients to receive sipuleucel-T (NCT00065442). The study primarily aimed at evaluate the overall survival compared to a placebo: it resulted 25.8 versus 21.7 months. Despite this positive outcome, the complex formulation and consequently the high cost of production of sipuleucel-T have hindered a more extensive diffusion. Nonetheless, sipuleucel-T has given a contribution to the development of numerous other DC-based vaccines. As various kind of DC vaccines are currently undergoing clinical trials for different cancer disease, many therapeutic candidates are envisioned, and the most relevant ones are listed in Table 1.

These studies highlight that these new strategies can become real possibilities in the fight against cancer, therefore they must be increasingly explored, as they can seriously improve and enhance the capabilities of existing cancer drugs.

## 7. EVs in Anti-Tumor Immunotherapy

The capabilities of the DCs as powerful and versatile APCs make them suitable to be the vehicles in cancer vaccines and anti-tumor immunotherapy. However, many drawbacks hinder their use in clinical treatments. DCs are a heterogeneous cellular population comprising various subtypes having different functional properties. Depending on the subset and on the received stimuli, DCs can display different capacities for antigen presentation, migration, and cytokine secretion. In particular, they can induce different T cell behaviors, by polarizing them into effector or tolerogenic cells [87]. Because of this heterogeneity they can thus promote either antitumor activity or regulation of immune tolerance, which is known to be a very limiting factor in vaccine success [88]. For example, some soluble immunosuppressive cytokines produced by tumor cells can convert immature DCs into tolerogenic DCs. These converted dendritic cells activate regulatory T (Treg) cells. Treg cells inhibit or downregulate proliferation of T cells such as CD4+ and CD8+, by promoting tumor proliferation [18]. Another limitation in the use of DCs is also due to their difficult storage aimed at maintaining their efficacy even for long periods of time. Finally, applying such therapies to a broad population is expensive and needs to meet rigorous quality control parameters. The exploitation of DC-derived exosomes (Dex) has proven to be a possible solution to the problems encountered in DC-based immunotherapy. Dex are characterized by unique molecular composition that allows them to maintain the immunostimulatory abilities of DCs. Indeed, Dex contain MHC-I and MHC-II molecules, which can respectively stimulate cytotoxic and helper T cells (T_h_ cells), together with costimulatory (CD86, CD40) and adhesion molecules (ICAMs), which can elicit strong immune responses toward cancer cells [89]. Furthermore, the lipid composition of exosomal membrane allows to storage Dex at −80 °C for more than 6 months maintaining high stability. Finally, compared to other types of cancer vaccines, cell-free treatment such as Dex-based treatment may be less vulnerable to immunotolerance and other immunomodulatory mechanisms that usually occur in tumors [90]. Some important preclinical studies carried out to evaluate the immunogenicity of Dex and their possibility of use in the production of new therapeutic vaccines are collected in Table 2.

Many Phase I clinical trials performed on various cancers have shown exosome-based vaccines to be safe, so exosomes could effectively represent a new approach for cancer immunotherapy. In particular, a Phase I clinical study investigated not only the feasibility and efficacy, but also the safety of administering autologous Dex loaded with peptides of melanoma-associated antigen (MAGE) to individuals with advanced NSCLC [96]. MAGE gene-derived peptides are widely expressed in many tumor and are able to stimulate antigen-specific CTL responses against MAGE-expressing cancer cells, resulting in tumor cell lysis. Therefore, thanks to their effectiveness in preventing and treating diverse cancers, MAGE has been used as a target for cancer immunotherapies [97]. In this Phase I trial, Morse et al. [96] isolated Dex by ultracentrifugation from peripheral blood mononuclear cells and loaded them with MAGE-A3, -A4, -A10, and MAGE-3DPO4 peptides. MAGE peptides were loaded directly into Dex after the purification step or indirectly into cultured DCs. The three patient groups in the clinical study were given the same Dex dose of 1.3 × 10^13^ MHC class II molecules in a volume of 3 mL once a week for 4 weeks, varying both the peptide loading method and the peptide concentration in each cohort. Dex therapy administered to 9 patients resulted non-toxic, tolerated and without the appearance of autoimmune responses. By contrast, In vitro immunologic analysis detected an increase in T cell activity in only one of tested patients, probably due to T_reg_-mediated suppression of immune cells. In 2/3 patients who had analyzable samples, an increase in T_reg_ was observed after the conclusion of Dex therapy. Other possible explanations were related to not well performing or low-sensitive assays, insufficient antigen presentation, or the absence of circulating antigen-specific T cells. Actually, it was hypothesized that might be due to activation of natural killer (NK) cells, that are cytotoxic lymphocytes involved in the innate immune system. NK cells are named “natural” because they recognize tumor or infected cells without the need of antigen-specific cell surface receptors or any preparatory activation and kill them by producing cytokines [98]. Confirming the hypothesis, Morse et al. [96] observed an increased activity of NK cells after immunization in two over four of the analysed patients, but in general, the clinical observations confirmed the successful immunization of the treated patients. Finally, the main clinical result was a very good disease stability, in both two patients with disease progression and in two initially stable patients. A similar study used MAGE peptides as cancer vaccine and was carried out in 15 patients with stage III/IV malignant melanoma [99]. Again, MAGE peptides were loaded directly into autologous DC-derived exosomes or indirectly into cultured DCs and administered to patients every week for 4 weeks. All patients underwent assessment of tumor status at 2 weeks after the fourth exosome vaccination. Vaccination was well-tolerated and resulted in a therapy response in four patients; in particular, a stabilization of the disease was proved in two patients receiving the highest dosage of directly-loaded exosomes. In contrast, one partial response and a minor response were identified in two patients who were then subjected to a continuation therapy, allowing for stabilization. Unfortunately, as in the previous study by Morse et al., no significant T cell response was observed.

Briefly, although there are clear advantages in Dex-based vaccine applications, not all treated patients show satisfactory responses due to the obstacles described above. Tumor-derived exosomes (Tex) are another type of exosomes investigated for the improvement of antitumor immune responses. Tex contain tumor-associated antigens expressed in the parental tumor cells and major histocompatibility complex (MHC) class I molecules [100]. Thus, Tex could present tumor antigens to DC and induce CD8+ T cell-dependent antitumor immune responses. In addition, tumor cells release a larger number of exosomes compared to healthy cells-derived exosomes. As a result, they are designated to become a new source of tumor antigens and thus a novel type of cell-free cancer vaccine [101,102]. However, previous studies reported that Tex can promote immune escape through different immunosuppressive mechanisms as described in the review by Whiteside et al. [103]. Generally, Tex are involved in the progression, regression and drug resistance of tumors and contribute to the development of metastases because they regulate immune responses by mediating communication between immune cells and cancer cells [104]. Many studies have verified the feasibility and functionality of Tex in activating immune responses against cancer in mouse models. Bu et al. [105] showed that a single dose of L1210 leukaemia-derive exosome-based vaccine not only inhibited tumor formation but also promoted protection against tumor growth in syngeneic mice. To assess the efficacy of the vaccine, the treated mice and the control group, consisting of unvaccinated mice, were stimulated with L1210 tumor cells 2 weeks after vaccination. Regarding tumorigenesis, 87.5% of the vaccinated mice were found to be tumor free, while in all untreated mice the tumor appeared in less than 10 days. Meanwhile, the rate of protection against tumor growth after 60 days was 85% in the vaccinated with a dose of 5 μg and 60% with the dose reduced to 2.5 μg. Finally, with regard to the immune response, CTL activity against L1210 was also observed and was better than the control group. Other studies on leukaemia vaccination proposed Tex as a possible antigen source for DC-based vaccination because of: Tex contain high amounts of tumor-associated antigens; Tex have markers that make easier the uptake by the DCs; the antigen presentation through MHC by DC induce CD8 T cell-dependent antitumor immune responses. Thus, a new perspective is emerging that is to produce cancer vaccines based on DCs loaded with exosomes derived from tumor cells, with the aim of exploiting the advantages deriving from the combined use of Tex and DCs. Indeed, Yao et al. [106] demonstrated that exosome-pulsed DCs induced stronger antitumor immunity than exosomes and DCs alone. Gu et al. have shown that In vitro, in the presence of DCs, it is possible to avoid inhibition of T_h_ lymphocytes activation by Tex and for this reason they have considered vaccination based on DC loaded with Tex (DC-TEX): they have proposed to load the DCs of patients with Tex circulating in the peripheral blood and to test the vaccine in a myeloid leukaemia mouse model [107]. In the end, they confirmed that Tex-pulsed DCs really increased the survival time of mice and determined a conspicuous activation of CTLs thanks to the combined use of Tex and DCs. Other studies that have evaluated the possibility of using extracellular vesicles as a vehicle in cancer vaccines are shown in Table 3.

**Figure 2:**
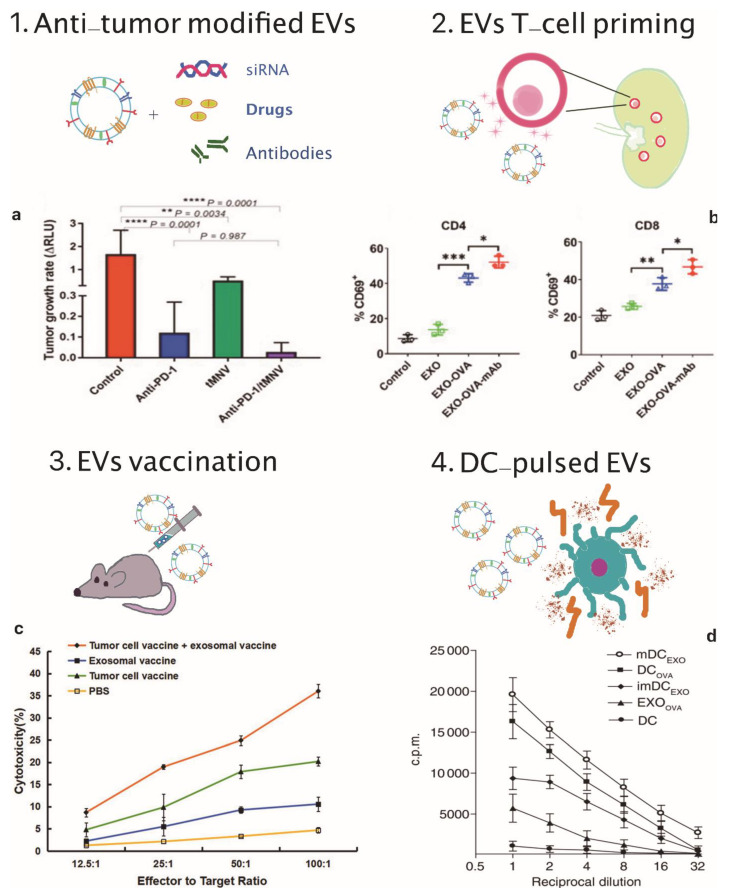
EVs applications in cancer immunotherapy. (**1**) Scheme of EVs as cargos of siRNAs, drugs and monoclonal antibodies and (**a**) therapeutic effect on tumor growth rate of anti-PD-1 and tMNV-directed therapy targeting B-catenin. Reproduced from [117] with the permission of Jhon Wiley and Sons. *p*-Values as indicated, one-way ANOVA analysis. (**2**) Scheme of EVs-mediated T-cell activation and (**b**) data analysis of CD69, a T-cell activation marker on CD4+ and CD8+ T cells following incubation with different exosomes formulations. Reproduced from [109] with permission of Elsevier. * *p* < 0.05, ** *p* < 0.01, *** *p* < 0.001, one-way ANOVA analysis. (**3**) Scheme of EVs vaccination and (**c**) tumor specific cytotoxic activity of the combination therapy involving exosomal vaccine and tumor cell vaccine against prostate cancer cells. Reproduced from [110] with permission of John Wiley and Sons. (**4**) Scheme of DC-pulsed EVs and (**d**) proliferative response of CD8+ T cells co-cultured with EXOOVA (10 μg/mL), DCOVA, mDCEXO and imDCEXO (3 × 10^4^ cells/well), determined by (3H)thymidine uptake assay after two days. Reproduced from [111] with permission of Jhon Wiley and Sons.

As described above, exosomes, and more broadly EVs, are also natural carriers of RNA and can be employed to deliver siRNA in silencing of genes for cancer treatment [112,113]. In addition, silencing of immunosuppressive genes via siRNA combined with immune checkpoint blockade therapy has been found to be a promising practice in new cancer immunotherapy applications [114,115]. As already mentioned, immune checkpoint blockade plays a key role in preventing the interruption of immune responses. Actually, immune checkpoint inhibitors, including monoclonal antibodies against programmed death 1 (PD-1), inhibit immunosuppressive molecules and restores the ability of the CTLs to kill cancer [116]. For all these reasons, Matsuda et al. used extracellular vesicles (EVs) for targeting β-catenin in hepatocellular carcinoma (HCC) via intrahepatic delivery of siRNA [117] β-catenin takes part in the signaling pathway of cell proliferation and often its alteration causes the development of carcinomas. In particular, Wnt/β-catenin pathway activation is associated with poor spontaneous T cell infiltration across most human cancers; thus, it contributes to immune escape [118,119]. By taking into account that responsiveness to anti-PD-1 therapy needs the presence of tumor antigen-specific T cells within tumor tissue, a poor or even absent T-cell infiltration can result in immune deserts and weak response to immunotherapy. Moreover, mutations in gene encoding β-catenin were identified among the most frequent alterations associated to the development of HCC [120,121]. Consequently, in this study, together with the β-catenin siRNA-loaded EVs, also anti-PD-1-based therapy were administered in order to reduce the tumor growth and at the same time, to improve the therapeutic action of immune checkpoint inhibitors. A synthetic model of hepatocellular cancer was induced in mouse livers by the co-expression of c-tyrosine-protein kinase Met (cMET) and mutant β-catenin via hydrodynamic injection (HDI) of DNA and plasmids [122]. The EVs were derived from bovine milk, which is a safe and scalable source of EVs [123]. First, the therapeutic effectiveness of anti-PD-1 was evaluated. Three weeks after HDI, two groups of mice bearing HCC were respectively administered with 250 μg/mouse of anti-PD-1 and with phosphate-buffered saline (PBS) for control measurements, for 2 weeks. The transfection of Gaussia luciferase (g-luc) and its expression level allowed to verify the success of the therapy against tumor growth in terms of relative luminescence units [124]. It was observed a reduced rate of tumor growth over a 6-week period and a very extended survival of the mice which received anti-PD-1 therapy, with a mean of 119 days compared to 96 in the control group. Secondly, to evaluate the efficacy of combined treatment with both therapeutic EVs and anti-PD-1 in targeting β-catenin, four groups of mice received different treatments three weeks after HDI for a period of 2 weeks. The first group received only 250 µg of anti-PD-1, the second group received only EVs (2 × 10^12^ particles/body), and the third group both anti-PD-1 and EVs. Last group was used as control and did not receive any treatment. Matsuda et al. [117] first demonstrated the In vitro effectiveness of β-catenin siRNA delivery via EVs: they incubated HepG2 cells with siRNA-loaded EVs and then found a decreased expression of β-catenin protein. Subsequently, they evaluated the effect of siRNA delivery in vivo. Tumor growth rate decreased with anti-PD-1, EV or both in the first three weeks after stopping treatment, but later a relapse was noted in 38% and 100% of the first and the second groups, respectively. Finally, the mice treated with both anti-PD-1 and EVs did not display any relapse, on the contrary they showed a considerable decrease in the tumor growth rate (between 3 and 12 weeks) even greater than that with either treatment alone. Therefore, this confirmed that targeting an oncogenic factor can improve the therapeutic effect of anti-PD-1. Ultimately, the combined treatment produced the highest degree of infiltration of CD8+ T cells within the tumor microenvironment. Furthermore, it has been confirmed that inhibition of β-catenin signaling in HCC improve the activation of specific T cells, promoting their infiltration into the tumor microenvironment and preventing CD8+ T-cell exhaustion following an initial response to anti-PD-1 therapy. However, there is still no scientific evidence to elucidate the mechanism used by β-catenin to promote CD8+ infiltration. Table 4 shows the currently active or already completed clinical trials involving EVs in immunotherapy. Figure 2 illustrates the main EVs application in cancer immunotherapy.

## 8. Conclusions

The research on extracellular vesicles has recently intensified considerably, leading to the achievement of important results in the application EVs for gene and drug delivery. In particular, exosomes have proved to be the ideal candidates to be used as therapeutic delivery vehicles. Based on this, the use of extracellular vesicles has also been extended to the field of cancer immunotherapy. A particular form of immunotherapy is represented by therapeutic anticancer vaccines. Among the different types of cancer vaccines investigated, the most promising are the cancer vaccines based on dendritic cells, as these cells facilitate the triggering of the immune response. Several clinical trials are currently active to evaluate the efficacy of therapeutic vaccines against various forms of cancer. As shown by numerous pre-clinical tests, dendritic cell-derived exosomes can also be used in the production of cancer vaccines. Clinical trials have confirmed the safety and feasibility of exosome-based cancer vaccines; however, some studies have not been fully satisfactory. In fact, vaccines are generally well tolerated by patients undergoing treatment, but often no significant immune response is observed. This indicates that significant progress has been made in building safe delivery vehicles but, at the same time, the clinical efficacy of extracellular vesicles based-cancer vaccines remains to be determined. The exosomes derived from tumor cells have been proposed as an alternative to the previous ones, as they are able to improve the antitumor immune responses. Nevertheless, some studies have shown that exosomes derived from tumor cells could be involved in processes that promote tumor proliferation, therefore, they are not preferable. To overcome these drawbacks, the possibility of producing cancer vaccines based on dendritic cells loaded with exosomes derived from tumor cells has recently emerged, but they are still under investigation. The use of extracellular vesicles in immunotherapy therefore seems to be hindered only by technological problems and not by qualitative ones. Therefore, an ever-increasing effort in this direction could lead to tangible results and above all to a new and innovative way to fight cancer.

## Figures and Tables

**Table 1:** Representative selection of the currently active clinical trials investigating dendritic cell-based cancer vaccines.

Condition	Treatment	Clinical Phase	NCT Identifier
Breast cancer	HER-2 pulsed DC Vaccine	Phase I	NCT02063724
Brain tumors	Autologous DCs pulsed with CSC Lysate	Phase I	NCT02010606
Prostate cancer	Autologous DCs loaded with mRNA from Primary prostate cancer tissue + hTERT + survivin	Phase I/II	NCT01197625
Sarcoma/soft tissue Sarcoma/bone sarcoma	DC vaccine + tumor lysate + imiquimod	Phase I	NCT01803152
Brain Metastases	Personalized cellular vaccine: tumor antigen mRNA-pulsed autologous DCs	Phase I	NCT02808416
Newly diagnosed glioblastoma	AV-GBM-1: autologous DCs loaded with autologous tumor antigens derived from self-renewing TICs	Phase II	NCT03400917
Multiple myeloma	ASCT + DC myeloma fusion vaccine + MAb CT-011 (pidilizumab)	Phase II	NCT01067287
AML	DC AML fusion vaccine	Phase II	NCT01096602
Advanced breast cancer	DCs co-cultured with CIK cells + capecitabine monotherapy	Phase II	NCT02491697

Legend: DC = dendritic cell, CSC = cancer stem cell, hTERT = human telomerase reverse transcriptase, TIC = tumor-initiating cell, ASCT = autologous stem cell transplantation, Mab = monoclonal antibody AML = acute myelogenous leukemia, CIK cells = cytokine-induced killer cells.

**Table 2:** Preclinical studies evaluating Dex immunogenicity for cancer vaccines.

Authors	Method	Main Outcomes	Refs
Théry C. et al.	In vitro	Dex can transfer functional peptide-loaded MHC class I and II complexes to DCs.	[91]
André F. et al.	In vitro and in vivo	Dex harbouring MHC class I/peptide complexes require DC for efficient priming of CTLs.	[92]
	In vivo	Dex mimic the capacity of mature DCs to initiate peptide-specific CD8+ T cell responses.	
Segura E. et al.	In vitro	Dex from immature DCs (imDC) and mature DCs (mDC) have different protein composition due to maturation signals. MHC class I molecules are up-regulated in mDC and reduced in mature exosomes. Molecules stimulating CD4 T cells are up-regulated in mDC and mature exosomes.	[93]
Sprent J.	In vitro	Peptide-pulsed Dex are immunogenic for CD8+ T cells also in the absence of APCs.	[94]
	In vivo	Peptide-loaded Dex induce high proliferative responses and CTLs induction, so priming CD8+ T cells.	
Viaud S. et al.	In vivo	Dex administration promotes proliferation, activation and cytotoxicity of NK cells.	[95]
	In vitro	Human Dex harbouring IL-15Rα lead to NK cell proliferation and IFNγ production	

**Table 3:** Preclinical studies investigating the use of EVs in cancer vaccines.

Therapeutic Agent	Condition	Outcome	Refs
Irradiated C6 glioma cell-derived MVs (IR-MVs)	Malignant C6 glioma	In vivo vaccination with IR-MVs promotes antitumor immune response leading to the apoptosis of glioblastoma cells and increases Th cells and CTL infiltration into the tumor.	[108]
DC-derived-exosomes functionalized with costimulatory molecules, MHCs, antigenic Ovalbumin peptide and anti-CTLA-4 antibody (EXO-OVA-mAb)	B16-OVA melanoma tumor model	Exosomes are targeted to T cells in vivo. EXO-OVA-mAb are able to effectively prime T-cell activation and proliferation, In vitro and in vivo. The fraction of memory T cells is increased in mice treated with vaccination. The antitumor efficacy is confirmed by the infiltration of both CD4 + and CD8 + cells and the CTLs/Treg ratio within the tumor site of vaccinated mice.	[109]
Interferon-γ-modified prostate cancer cell- derived exosomes	RM-1 prostate cancer	Vaccine induces macrophages differentiation and the production of antibodies, reduces tumor angiogenesis and metastasis rate, inhibits tumor growth and prolongs survival time of mice with metastatic prostate cancer.	[110]
Interferon-γ-modified prostate cancer cell- derived exosomes + IFN-γ-modified RM-1 cell vaccine	RM-1 prostate cancer	Exosomal vaccine improves the T cell response generated by the tumor cell vaccine and downregulates in the expression of IDO1 and PD-L1 immune checkpoints. Combination therapy show the highest tumor-specific cytotoxic activities compared to vaccine monotherapies and tumor growth is significantly suppressed.	[110]
Mature DCs pulsed with ovalbumin protein-pulsed DC-derived exosomes (EXO-pulsed DCs)	B16-OVA melanoma tumor model	EXO-pulsed DCs stimulate CD8+ T-cell proliferation and differentiation into CTL effectors In vitro and in vivo. EXO-pulsed DCs induce stronger immunity against lung tumor metastases and can eradicate established tumors. They also induce strong long-term OVA-specific CD8+ T-cell memory	[111]

**Table 4:** Collected currently active or completed clinical trials investigating the use of EVs-based immunotherapies.

Condition	Treatment	Year	Clinical Phase	NCT Identifier and References
Advanced NSCLC	Dex loaded with the MAGE tumor antigens	2005	Phase I	[96]
Metastatic melanoma	Autologous exosomes pulsed with MAGE 3 peptides	2005	Phase I	[99]
Colorectal cancer	Ascites-derived exosomes (Aex) in combination with GM-CSF	2008	Phase I	[125]
Melanoma	Human Dex bearing NKG2D ligands	2009	Phase I	[95]
NSCLC	Tumor Antigen-loaded Dex	2010	Phase II	NCT01159288
Unresectable NSCLC	IFN-γ-Dex loaded with MHC class I- and class II-restricted cancer antigens	2015	Phase II	[89]

NSCLC = non-small-cell lung cancer, IFN = interferon γ, GM-CSF = granulocyte–macrophage colony-stimulating factor.
